# Action and perception in social contexts: intentional binding for social action effects

**DOI:** 10.3389/fnhum.2014.00667

**Published:** 2014-09-02

**Authors:** Roland Pfister, Sukhvinder S. Obhi, Martina Rieger, Dorit Wenke

**Affiliations:** ^1^Department of Psychology III, Julius Maximilians University of WürzburgWürzburg, Germany; ^2^Department of Psychology, Wilfrid Laurier UniversityWaterloo, ON, Canada; ^3^Institute for Psychology, UMIT, University for Health Sciences, Medical Informatics and TechnologyHall in Tirol, Austria; ^4^Department of Psychology, Max-Planck-Institute for Human Cognitive and Brain SciencesLeipzig, Germany; ^5^Department of Psychology, Humboldt University at BerlinBerlin, Germany

**Keywords:** intentional binding, action effects, social actions, action and perception, sense of agency

## Abstract

The subjective experience of controlling events in the environment alters the perception of these events. For instance, the interval between one's own actions and their consequences is subjectively compressed—a phenomenon known as intentional binding. In two experiments, we studied intentional binding in a social setting in which actions of one agent prompted a second agent to perform another action. Participants worked in pairs and were assigned to a “leader” and a “follower” role, respectively. The leader's key presses triggered (after a variable interval) a tone and this tone served as go signal for the follower to perform a keypress as well. Leaders and followers estimated the interval between the leader's keypress and the following tone, or the interval between the tone and the follower's keypress. The leader showed reliable intentional binding for both intervals relative to the follower's estimates. These results indicate that human agents experience a pre-reflective sense of agency for genuinely social consequences of their actions.

## Introduction

The physical world is quite simple, at least when considering how it can be affected by one's own actions: Pressing a light switch in a dark hallway will turn on the light just as reliably as jumping into a puddle will make some water splash around. In other words: Every action an agent chooses to perform will produce certain effects in the environment, and these effects can be predicted with ease in many cases. Actively bringing about an action effect in the environment gives rise to *sense of agency*, the subjective experience of controlling one's actions and, through them, events in the outside world (Haggard and Tsakiris, 2009). A major precondition for sense of agency to arise is a high contingency between actions and following effects (Metcalfe and Greene, 2007; Moore et al., 2009a). As the above examples show, the physical world offers almost ideal preconditions for feeling control over various ensuing events, while at the same time being able to tell which events escape one's own influence.

By contrast, matters become more complicated when considering social consequences of own actions: Human actions often aim at changing the behavior of another agent, and in this situation, the action's exact effects do not only depend on the action itself but also on how the other agent actually responds to it. Interestingly, sense of agency has not yet been studied for actions that explicitly aim at influencing another agent's behavior. The present study therefore addressed this issue by measuring a specific, pre-reflective component of sense of agency that is known as *intentional binding* (Haggard et al., 2002). Before describing these experiments, we give a brief overview of different measures of sense of agency and of previous studies on sense of agency in social contexts that involved two individuals jointly producing a given effect.

### Measuring sense of agency

Sense of agency can be measured directly and indirectly (Haggard and Tsakiris, 2009). Direct measures of sense of agency are usually obtained via self-reports in terms of judgments of agency on a predefined rating scale (e.g., Wegner et al., 2004; Sato and Yasuda, 2005; Wenke et al., 2010). Obviously, these measures draw on reflective aspects of sense of agency that are available to introspection. Thus, they have often been viewed as capturing mainly processes of retrospective inference which compare the match between current intention and an experienced effect (e.g., Wegner, 2003).

Indirect measures, by contrast, aim to assess pre-reflective correlates of agency, and the phenomenon of intentional binding is one of these correlates (for an overview of indirect measures, see Haggard and Tsakiris, 2009). Intentional binding refers to the finding that the perceived time interval between voluntary actions and ensuing perceptual events is subjectively compressed (Haggard et al., 2002; Moore and Haggard, 2008; but see Buehner and Humphreys, 2009; Buehner, 2012). It has been argued that intentional binding strongly depends on pre-reflective processes that do not require self-referential processing. In particular, intentional binding was suggested to reflect the low-level sensorimotor basis of sense of agency (Moore and Obhi, 2012) and might primarily reflect what Synofzik et al. (2008) refer to as “feelings of agency.” Compared to explicit self-report judgments, indirect measures of sense of agency such as intentional binding are assumed to be less affected by prior beliefs about who is in control (but see Desantis et al., 2011). Therefore, intentional binding and explicit agency judgments seem to capture at least partly different processes and might yield diverging results in some situations (Ebert and Wegner, 2010; for a review, see Moore and Obhi, 2012). This is not to say however that intentional binding depends on predictive processes alone. Previous studies have shown that intentional binding depends on both, efferent motor prediction and retrospective inference that occurs right after an agent experiences a certain effect to result from his or her action (Moore and Haggard, 2008; Moore et al., 2009a). Thus, our use of the term “pre-reflective” aims at distinguishing processes that are captured by indirect measures rather than by direct self-reports, without implying a particular interpretation in terms of predictive or retrospective mechanisms.

### Sense of agency in social interaction

Previous studies on sense of agency in social interaction focused on settings that were explicitly designed to be highly ambiguous about which of two agents had caused a certain event. Such ambiguous settings allow investigating whether agents may attribute authorship for an event to themselves even if this event was actually caused by someone else. And indeed, such “vicarious” agency has been demonstrated in different experimental contexts (Nielsen, 1963; Wegner and Wheatley, 1999; Wegner et al., 2004). For instance, Wegner and Wheatley (1999) asked two actors to perform a joint (mouse) movement and each actor could stop the mouse cursor at a time of his or her choosing. After stopping, participants provided direct judgments of agency about the stopping action. Interestingly, they reported a high degree of agency even when the other actor had actually stopped the movement, provided that the effect of the stopping action (the mouse cursor resting on a particular object on the screen) corresponded to an auditory prime naming that object prior to the action.

In addition to introspective self-reports of sense of agency, Obhi and colleagues recently suggested that sense of agency in social situations may also include pre-reflective processes as measured via intentional binding (Strother et al., 2010; Obhi and Hall, 2011a,b). Similar to an experiment by Wegner and Wheatley (1999), the participants of Obhi and Hall (2011a) jointly engaged in a task (pressing the space bar on a computer keyboard), which in turn produced a joint effect (a tone). Both participants placed their index finger on one end of the space bar and were encouraged to press the key at a time of their choosing. If the other participant initiated the keypress first, they were to join in and press the space bar down as well. In addition to explicit judgments of agency, these authors also assessed intentional binding and found reliable binding effects for both, self-initiated and other-initiated actions. Interestingly though, explicit self-reports of agency differed, such that only those individuals who actually initiated the key press reported being responsible for the outcome. Overall, these results suggest that intentional binding might not be restricted to own actions. Instead, it might also occur for another person's actions, at least when agents jointly produce an effect that matches the individual's intention.

### Controlling other people: the present experiments

In the present experiments, we investigated sense of agency in a different social situation: Rather than creating ambiguity about who had caused a certain effect in the environment, we set up a situation in which one of two agents clearly was the “leader” and prompted a second participant, the “follower,” to carry out an action. That is, both agents performed their own distinctive actions *with the action of the follower being triggered by the leader action*. In fact, such situations are very common in everyday interactions. For example, someone might ask another person to open a window or, in organizational settings, a person higher up in the hierarchy might prompt his or her subordinates to carry out a certain task. In such situations, the leader clearly affects the follower's action although, of course, the follower is immediately responsible for initiating and performing it. As outlined above, the follower's action is not as predictable as action effects in the physical world tend to be, neither in terms of timing (contiguity) nor in terms of actual occurrence (contingency). It is thus unclear, whether pre-reflective components of sense of agency—as measured via intentional binding—arise for such social action effects. Finding intentional binding for the leader regarding the follower's action would indicate that the representation of the follower's action potentially affected low-level predictive motor processes, similar to situations in which one's own action causes predictable effects in the physical environment (Haggard et al., 2002).

**Figure 1:**
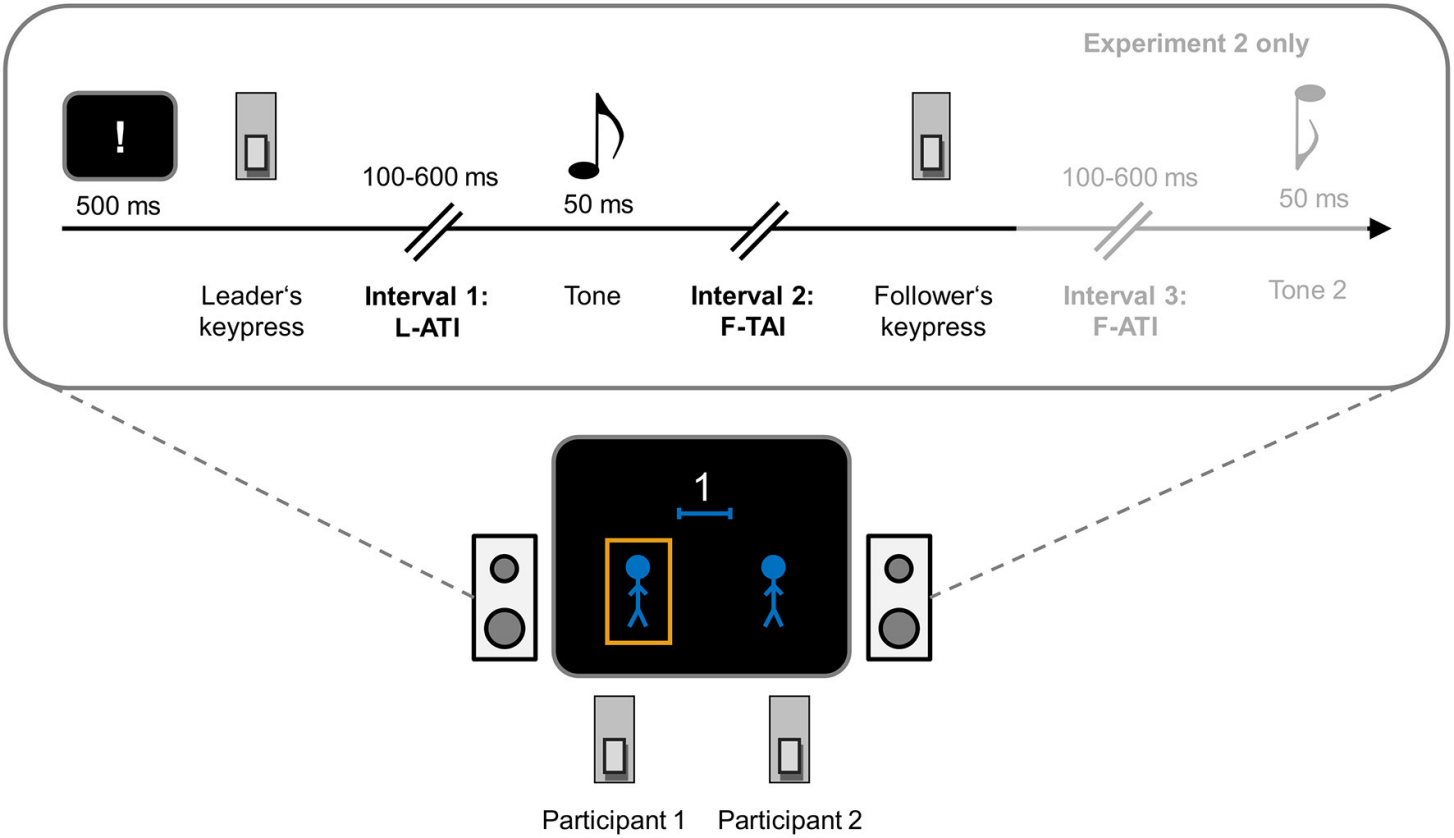
**Setup and trial procedure of Experiment 1 and 2**. Participants sat next to each other and operated one response key each. One participant acted as the leader of an action sequence whereas the other participant acted as the follower: The leader initiated the action sequence at leisure after an exclamation mark had disappeared from the computer screen. The leader action triggered an effect tone after a variable action-effect interval of 100–600 ms. This tone also served as a go signal for the follower. The follower's action triggered an effect tone in Experiment 2, but not in Experiment 1. At the end of the trial, either the leader or the follower had to estimate the duration of either the leader's action-tone interval (L-ATI), the interval between the tone and the follower's action (F-TAI), or (Experiment 2 only) the follower's action-tone interval (F-ATI).

Supporting evidence for this speculation comes from a recent study on the role of anticipated social action effects for effect-based action control (Pfister et al., 2013). In this study, two participants also worked on a task in which one of them was the designated leader and the other was the designated follower. The leader performed a long or short keypress in response to an imperative stimulus on a computer screen that only he or she was able to see. In different blocks, the follower either imitated the leader's action (e.g., performing a short keypress in response to a short keypress of the leader), or counter-imitated the leader's action (performing a long keypress in response to a short keypress of the leader). The leader showed better performance, i.e., faster responses, in the imitation condition as compared to the counter-imitation condition. Because the follower's imitation or counter-imitation response only occurred after the leader action, these findings indicate that anticipated changes of the follower's behavior affected the leader's action planning. The results of Pfister et al. (2013) thus suggest that social action effects may indeed become integrated in action control. This, in turn, might give rise to intentional binding for these effects (for additional comments on effect-based action control in social settings, see Ray and Welsh, 2011; Pfister et al., 2014). We tested this prediction in two experiments in which two participants acted interdependently in a simple action sequence (see Figure 1).

One participant, the leader, started the action sequence by pressing a key. After a variable interval, an effect tone was presented which served as a go signal for the follower to also press a key. The interval between the leader's keypress and the onset of the effect tone, termed the leader's action-tone-interval (L-ATI), as well as the interval between the tone and the follower's keypress, termed follower's tone-action-interval (F-TAI; i.e., his or her response time) were estimated by both, leader and follower (for previous uses of interval estimation tasks in research on temporal binding, see Engbert and Wohlschläger, 2007; Engbert et al., 2007, 2008; Moore et al., 2009b; Wenke and Haggard, 2009; Humphreys and Buehner, 2010). Intentional binding for the leader would become evident in terms of giving shorter interval estimates than the follower. Based on the above argument, we expected to observe intentional binding for the leader not only for his or her action effect in the physical environment (the action-contingent tone), but also for the ensuing action of the follower.

## Experiment 1: intentional binding for social action effects

In Experiment 1, we investigated whether different roles in a social setting, i.e., being a leader or a follower, results in different representations of the actions conducted in this setting. Specifically, we were interested in whether the leader, whose action prompted the follower to react, would represent the follower's action in the same way the leader represents other effects of the own action. To investigate whether the representation of such action effects is mirrored in indirect measures of sense of agency, we assessed intentional binding in terms of direct interval estimates (see also Figure 1). The to-be-estimated interval on a given trial was pre-specified, whereas a cue at the end of the trial indicated who was to judge. We expected intentional binding for the leader's effect tone to be mirrored in shorter interval estimates for the leader estimating the L-ATI than for the follower estimating the L-ATI. Further, if the follower's response is also coded as an additional action effect for the leader, the leader should perceive this event to occur earlier in time than the follower, giving rise to shorter estimates for the F-TAI, too. We thus predicted shorter interval estimates by leaders than by followers, not only with regard to the first interval (L-ATI) but, more importantly, also for the second one (F-TAI).

### Methods

#### Participants

Twenty-eight volunteers from the city of Leipzig were paid for participation (8 males; all right-handed; mean age = 22.9 years). All participants reported normal or corrected-to-normal vision and hearing and were naive as to the purpose of the experiment. The two participants of each session were of the same gender. The study was conducted in accordance with the Declaration of Helsinki and the procedures were approved by the local ethics committee.

#### Material, apparatus, and procedure

The two participants of each pair worked together in front of a 17” monitor and operated one response key each with their right hand. The keys were mounted safely on the table and were connected to the computer via the parallel port. A second monitor was turned sideways to the experimenter and could not be observed by the participants.

Participants received written instructions and were told that their task was to estimate the length of either of the two intervals (in ms) and that the to-be-judged interval (i.e., either L-ATI or F-TAI) was constant for each block of trials. They were assigned to the roles of leader and follower and were informed that their roles would change after the first half of the experiment. Across participant pairs, we counterbalanced whether the left- or right-sitting participant started as leader.

Figure 1 shows a schematic of an experimental trial. Each trial started with the presentation of a white exclamation mark in the center of the screen (20 pt Arial font). After a delay of 500 ms, the exclamation mark disappeared and the program waited for the leader's key press. Leaders pressed their key at a time of their choosing. The leader's key press triggered an effect tone that appeared after a random action-tone interval between 100 and 600 ms drawn from a uniform distribution. This was done in order to match typical RTs of previous experiments with comparable settings (Engbert et al., 2007, 2008). However, participants were told that the interval varied between 1 and 1000 ms, and they were similarly informed that typical reaction times are in the range of up to 1000 ms. Sinusoidal tones with a duration of 50 ms and a frequency of 400 and 800 Hz served as auditory action effects. Tones were presented via two loudspeakers that stood to the right and to the left of the monitor, and the pitch of the tone depended on the key that was pressed. Because each pair of participants had a fixed sitting order and only operated one key each, this implied that the tones were participant-specific for the duration of the entire experiment. The assignment of tones to keys was counterbalanced across participant pairs.

The tone served as go-signal for the follower and the program waited for a maximum of 1000 ms for the follower's key press. Then, after an additional SOA of 500 ms, the judgment screen was presented (see Figure 1). The judgment screen consisted of two matchstick men and either the left or the right matchstick man was marked by an orange box to indicate the judge in the current trial. That is, participants only learned at the end of each trial whose turn it was to judge the interval, to ensure that both participants always paid attention to the events at all times. Additionally, the judgment screen contained a number above the matchstick men that reminded participants which interval to judge (“1” for the L-ATI, “2,” for the F-TAI). The to-be-judged intervals were blocked such that participants knew in advance which interval to focus on. The order of to-be-judged intervals was counterbalanced across participant pairs, but remained constant for the two experimental halves for each pair.

Participants gave their interval judgments orally, and the experimenter noted the time estimates and initiated the next trial. Anticipations (leader actions before the exclamation mark disappeared, follower reactions before tone onset), omissions (follower's reaction time > 1000 ms), or wrong order of keystrokes (follower before leader), triggered a warning message on the screen and the next trial started afterward. These trials were removed from the analyses.

Each experimental half started with three different practice blocks that allowed participants to familiarize themselves with the task in each role (leader, follower). The first practice block comprised 10 trials in which participants only had to press the keys in the correct order without estimating the interval length. This block was followed by two additional training blocks of 20 trials (each pertaining to one of the intervals) during which the participants were instructed to make interval estimates. In these two training blocks, the experimenter gave vague feedback about the judgments by classifying the to-be judged intervals as short, medium, and long (and shorter/longer than the previous interval). To this end, the actual interval length was displayed on the experimenter's monitor throughout the experiment. Data of the training blocks were not analyzed.

After the training blocks, two test blocks of 20 trials each were performed for each interval (totaling to 20 interval estimates for each combination of interval and judge). Both blocks relating to a specific interval immediately followed each other and the sequence of intervals matched the sequence of the training blocks. Thus, if the participants judged the L-ATI in the first training block and F-TAI in the second training block, they started with two blocks of L-ATI judgments and continued with two blocks of F-TAI judgments. The interval sequence was counterbalanced across participant pairs. The fourth test block marked the end of the first half of the experiment and was followed by a longer break before participants continued with changed roles.

### Results

The first trial of each block and trials with errors (4.3%) were excluded from data analysis. For the remaining test trials, we computed binding scores by subtracting the actual interval length from the respective interval estimate; negative binding scores thus indicate a subjective compression of the interval. These binding scores were then subjected to an outlier correction for each participant and condition (|z| > 2.5; 1.0%).

Preliminary analyses examined the correlation of binding scores and actual interval lengths across all trials for each participant. These correlations were submitted to a Fisher-Z transformation, averaged across participants, and re-transformed afterward. This analysis yielded a strong mean correlation indicating more pronounced binding for longer intervals, *r* = 0.60, with the mean Z-value differing significantly from zero, *t*_(27)_ = 11.28, *p* < 0.001. Such a correlation might introduce potential confounds to any analysis of the raw binding scores because, unlike in most previous studies, our design did not allow for matching interval lengths across all conditions. This potential confound becomes evident when considering a session in which the particular follower responds very slowly as compared to most other participants: Such a slow response time (that cannot be manipulated experimentally) might be sufficient to introduce various biases in the obtained interval estimates from both participants and thus distort the pattern of results. We therefore decided to perform an analysis of regression residuals instead of analyzing the raw binding scores, even though the raw binding scores yielded a similar pattern (with profound underestimation for all conditions except for the follower estimating the F-TAI, see Table A1 in the Supplementary Material).

Such analyses of regression residuals are performed in two steps (cf. Maxwell et al., 1985; Pfister, 2011). In the first step, we calculated a linear regression for each individual participant to estimate the impact of the interval length on binding scores (irrespective of the experimental condition). The scores predicted by the regression analysis were then compared to the actual binding scores to calculate the regression residuals, i.e., the portion of the interval estimate that could not be accounted for by the interval length itself. We then submitted the mean regression residuals to a 2 × 2 repeated-measures ANOVA with the factors judge (leader vs. follower) and to-be-judged interval (L-ATI vs. F-TAI).

**Figure 2:**
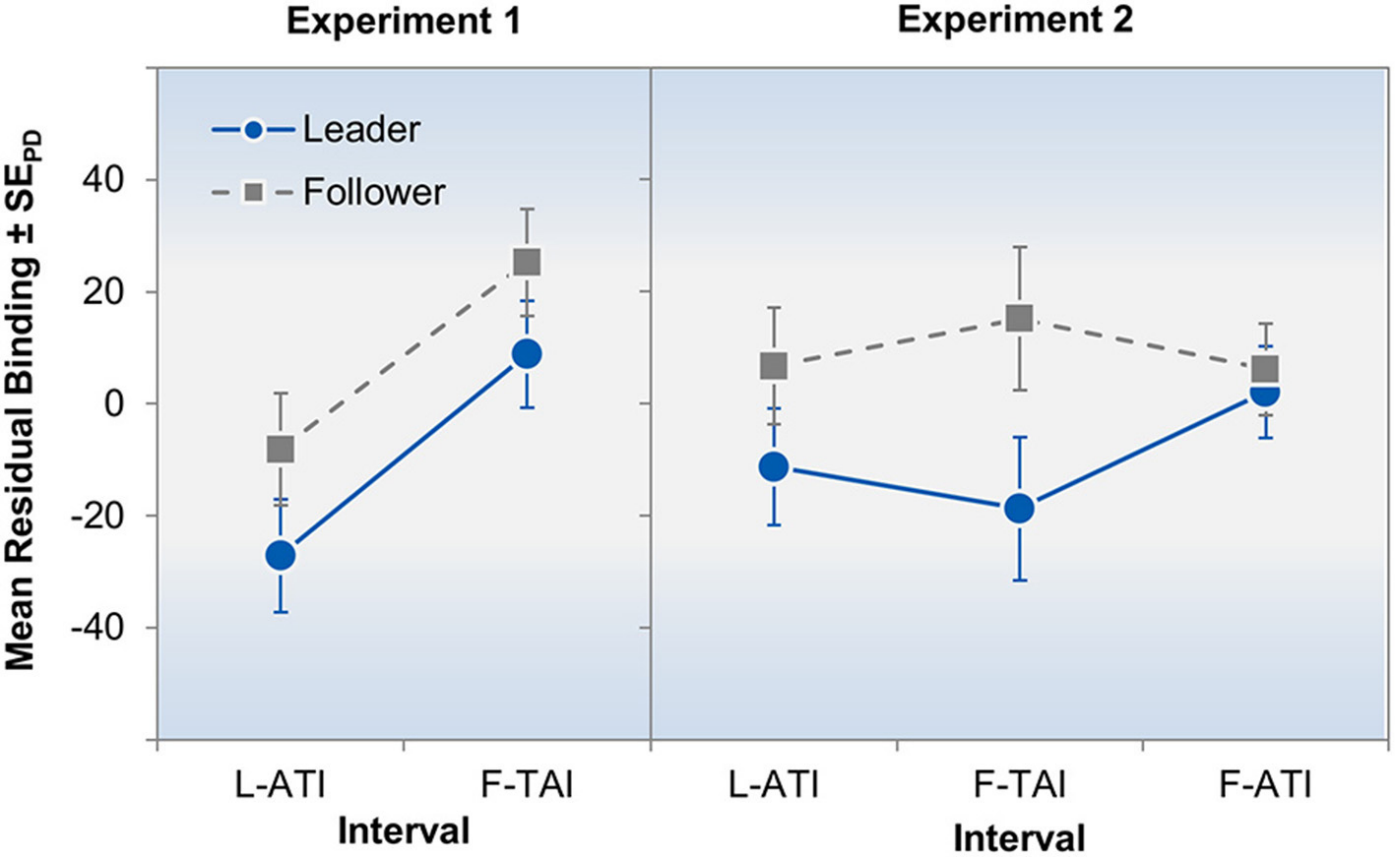
**Results of Experiment 1 (left panel) and Experiment 2 (right panel)**. The plots show the mean regression residuals for the leader's action-effect-interval (L-ATI), the interval between the leader's tone and the follower's action (F-TAI), and the follower's action-tone interval (F-ATI, Experiment 2 only). Negative values indicate shorter estimates and error bars represent standard errors of paired differences (SE_PD_; Pfister and Janczyk, 2013), computed separately for each interval.

As hypothesized, participants gave shorter interval estimates for both intervals when acting as leader than when acting as follower (Figure 2, left panel), *F*_(1, 27)_ = 7.65, *p* = 0.010, η^2^_p_ = 0.22. Additionally, the first interval was consistently judged to be shorter than the second interval by both judges, *F*_(1, 27)_ = 16.30, *p* < 0.001, η^2^_p_ = 0.38. Both main effects were additive as indicated by a non-significant interaction (*F* < 1). Considered separately, one-tailed *t*-tests showed significant differences between leaders and followers both, for the L-ATI, *t*_(27)_ = 1.89, *p* = 0.035, *d* = 0.36, and the F-TAI, *t*_(27)_ = 1.72, *p* = 0.048, *d* = 0.33.

### Discussion

Experiment 1 investigated intentional binding for social action effects, i.e., responses of another agent. The results indicate that the leader of the action sequence did experience intentional binding for the follower's action. This finding is especially striking in light of previous research on the perceived timing of observed actions that are not performed in response to own actions (as in the present setup) but rather, independently of any other agent (Wohlschläger et al., 2003a,b). These previous studies found either no difference between the estimated onsets of own and observed actions, or even the reverse pattern, with observed actions being judged to occur later in time than own actions. The present setting thus clearly did not only compensate for this bias but shifted the pattern of estimates toward an underestimation of the follower's tone-action interval by the (observing) leader, as compared to the follower him- or herself. It thus seems as if pre-reflective components of sense of agency do indeed occur for social action effects even despite the challenges that come with the social setting.

On closer inspection, however, the design of Experiment 1 seems to lack a critical feature that is often present in real-world interactions outside the laboratory. For instance, when asking someone to open a window, the “follower” clearly achieves an action effect (i.e., the opened window), rather than simply performing a particular movement as was the case in Experiment 1. Experiment 2 thus introduced an additional component to the task: The follower's action now triggered a tone as well, and we obtained interval estimates for this interval in addition to the two intervals of Experiment 1. This setting thus provided the opportunity to replicate the central results of Experiment 1 (stronger F-TAI binding for leaders than for followers) while at the same time probing for differential binding for the third interval for the leader and the follower.

## Experiment 2: investigating the follower's action effect

Experiment 2 extended the action sequence of Experiment 1 so that the follower now also produced an effect tone by her or his keypress and this tone differed in pitch from the leader's tone. As for the leader's effect tone, the follower's effect occurred after a variable interval and we label this interval the F-ATI. Participants either estimated the length of the L-ATI, the F-TAI, or F-ATI. Our main question was whether the results of Experiment 1 would replicate in this setting and whether the stronger intentional binding for leaders compared to followers would also transfer to the additional F-ATI.

This question is related to previous studies that found vicarious agency for the action effects of others in ambiguous situations (Wegner and Wheatley, 1999; Strother et al., 2010; Obhi and Hall, 2011a,b). In the present setup, however, it was clear that the follower ultimately triggered his or her effect tone. For such observed actions, it is not clear whether or not binding occurs, with some studies suggesting a negative answer (Engbert et al., 2007, 2008) and others suggesting a positive one (Obhi and Hall, 2011a, Experiment 2; Poonian and Cunnington, 2013; cf. also Buehner and Humphreys, 2009).

As a manipulation check, we further wanted to assess how leaders and followers conceptualized their own and the other agent's actions. To this end we developed and administered an *ad-hoc* questionnaire that was loosely based on action identification theory (Vallacher and Wegner, 1987, 1989) According to this theory, agents may construe own actions on different levels of goal-directedness, by either focusing on immediate movements or, alternatively, on more distal goals. Similarly, we aimed at assessing whether the participants of Experiment 2 construed the situation in terms of the responses or any of the corresponding action effects.

### Methods

#### Participants

Twenty-four volunteers were paid for participation (8 males; all right-handed; mean age = 23.7 years). They fulfilled the same criteria as in Experiment 1. All but one participant reported normal or corrected-to normal vision and hearing; the remaining participant reported an otitis of the middle ear in the second session and we therefore did not analyze his data.

#### Material, apparatus, and procedure

Experiment 2 employed the same design as Experiment 1 (see Figure 1) with the following modifications. In each trial, the follower's reaction produced a tone after a variable interval of 100–600 ms (uniformly distributed). Participants were told that both, the leader's and the follower's action-tone interval varied between 1 and 1000 ms. Participants started with a training block of 12 trials without interval estimates. Next, they underwent three blocks per to-be-judged interval—the L-ATI, F-TAI, and the F-ATI—whereas the order of intervals was counterbalanced across participant pairs. Each block consisted of 24 trials and the first block of each triplet served as a training block for the respective interval. The roles of leader and follower were still constant throughout one half of the experiment, but the two halves were now held as separate sessions on two successive days. At the end of each session, participants completed an *ad-hoc* questionnaire targeting their perception of the task (see the below).

#### Post-experimental questionnaire

Each participant of Experiment 2 judged leader and follower actions after both sessions. If the participant had been the leader in a session, he or she completed the questionnaire in the leader role (“How would you describe your own action?” and “How would you describe the follower's action?”), and if the participant had been the follower, he or she completed the questionnaire in the follower role. For each rating, they had to choose one out of six descriptions which best matched their perception of the leader and, separately, the follower role. The six items of the questionnaire described the actions as (1) *finger movement*, (2) *key press*, (3) *producing a signal for the follower* (leader) or *reacting to the leaders signal* (follower), (4) *starting an action sequence* (leader) or *finishing an action sequence* (follower), (5) *producing a tone*, and (6) *none of the above*. For follower actions, these items were ordered as described above, whereas for leader actions, the order was 1, 2, 5, 3, 4, and 6.

### Results

#### Residual binding scores

The analysis followed the same strategy as for Experiment 1 and all test trials with errors (3.1%) were excluded from data analysis. The remaining trials were outlier-corrected (|z| > 2.5; 1.1%) and entered a linear regression to estimate regression residuals. The analysis of regression residuals was again motivated by a substantial correlation of interval length and binding scores, *r* = 0.49, *t*_(22)_ = 11.74, *p* < 0.001 (see Table A1 in the Supplementary Material for the raw data). Residuals were submitted to a 2 × 3 repeated-measures ANOVA with the factors judge (leader vs. follower) and to-be-judged interval (L-ATI vs. F-TAI vs. F-ATI) and we used the multivariate approach to repeated-measures ANOVA to counter possible violations of sphericity.

The right panel of Figure 2 shows the mean residual binding scores for all design cells. Leaders again perceived the intervals to be shorter than followers, *F*_(1, 22)_ = 8.02, *p* = 0.010, η^2^_p_ = 0.27, and this effect was qualified by a marginally significant interaction, *F*_(1, 21)_ = 2.65, *p* = 0.094, η^2^_p_ = 0.20. The main effect of interval did not approach significance (*F* < 1). The interaction was driven by manifest differences between leader and follower for the L-ATI, *t*_(22)_ = 1.73, *p* = 0.049, *d* = 0.36, and the F-TAI, *t*_(22)_ = 2.65, *p* = 0.007, *d* = 0.55, but not for the F-ATI, *t*_(22)_ = 0.49, *p* = 0.313, *d* = 0.10, as indicated by one-tailed *t*-tests.

Furthermore, leaders and followers differed slightly regarding the judgments of their “own” tone: Residual binding scores for leaders judging the L-ATI (−11 ms) were marginally significantly lower than residual binding scores of followers judging the F-ATI (−6 ms), *t*_(22)_ = 1.92, *p* = 0.068, *d* = 0.040, thus indicating an asymmetry in intentional binding when intervals of the same type were compared.

#### Questionnaire data

**Figure 3:**
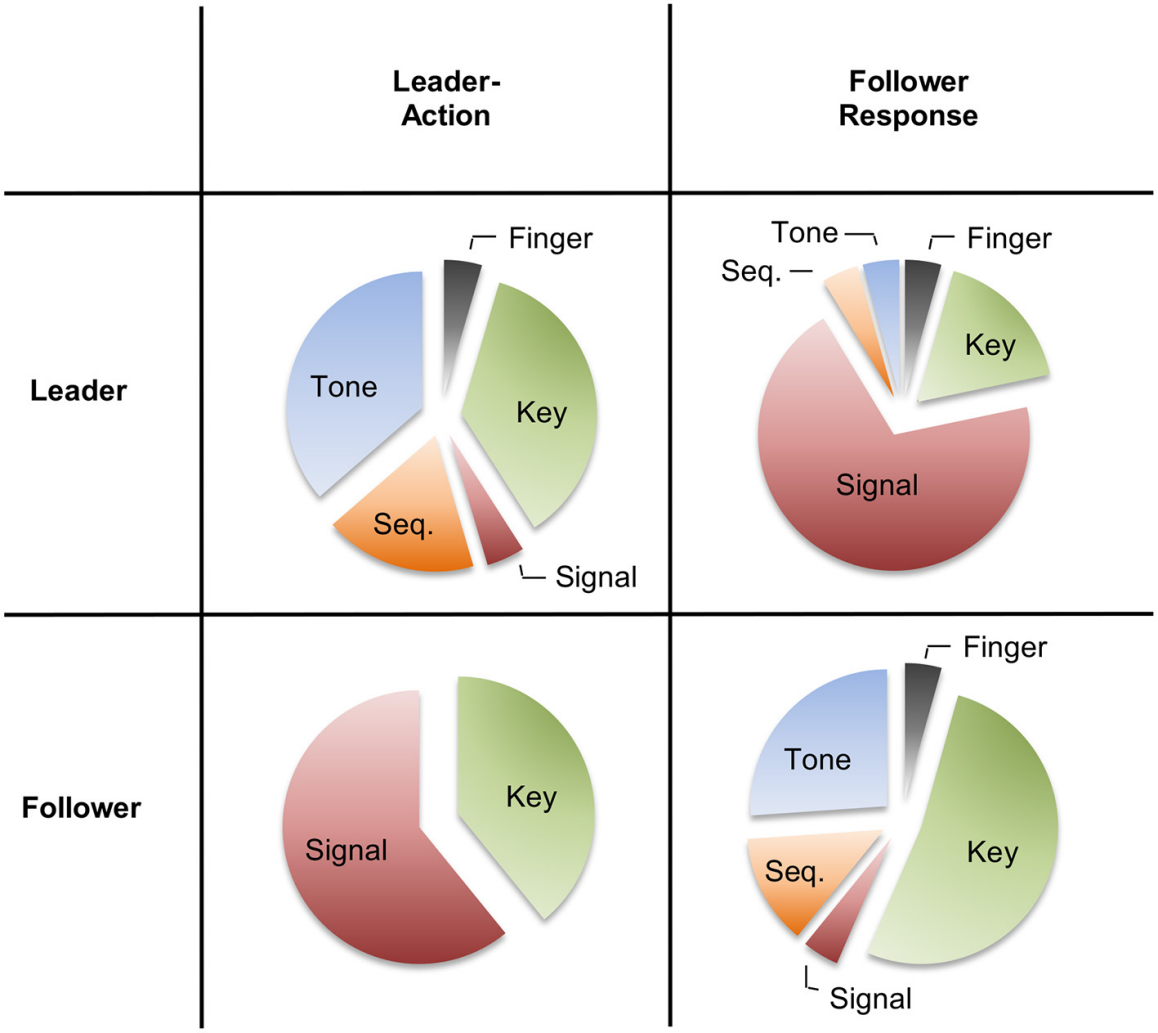
**Responses in the post-experimental questionnaire as judged from the leader's perspective (top row) and the follower's perspective (bottom row)**. The available answers to describe the respective actions were (1) *finger movement*, (2) *keypress*, (3) *signaling response/reacting to the signal*, (4) *initiating/completing an action sequence*, (5) *producing a tone*, and (6) *none of the above (chosen only once; not displayed)*.

The main questionnaire results are shown in Figure 3. Interestingly, the participants' judgments mainly depended on whether they judged their own role or the role of the other participant, irrespective of the role itself. For observed actions—leader actions from the follower perspective and follower actions from the leader perspective–, participants mainly used the labels of keypresses (2) and, crucially, the task-related description of signaling a response or responding to the signal (3). The tendency toward this latter description was especially pronounced for leaders, suggesting that they indeed construed the follower response as an effect of their preceding action. For performed actions—leader actions from the leader perspective and follower actions from the follower perspective—participants mainly used the labels of keypresses (2) and of producing a tone (5). Again, this latter option was chosen more often by leaders than by followers.

These impressions were confirmed by Cochran's Q-tests across the four conditions (leader judging the leader action, leader judging the follower action, follower judging the leader action, and follower judging the follower action). A separate test was conducted for each possible answer (1–6), when coding the presence of this answer as 1 and any other option as 0 for each condition. These tests showed significant between-conditions differences for answer 3 (“signal”), *Q*_(3)_ = 34.94, *p* < 0.001, and answer 5 (“tone”), *Q*_(3)_ = 15.34, *p* = 0.002, indicating that these options differed in frequency across the four conditions. Further, a marginally significant effect emerged for answer 2 (“keypress”), *Q*_(3)_ = 6.44. *p* = 0.092), whereas the remaining answers were distributed equally across the conditions (*p*s > 0.141).

### Discussion

Experiment 2 replicated the findings of Experiment 1 regarding the leader's intentional binding for the own action effect and the follower's action in terms of reduced interval estimates for both intervals. Additionally, the results of Experiment 2 did not yield any differences in intentional binding regarding the follower's action effect. More precisely, neither the leader nor the follower showed any indication of a subjective compression of the F-ATI. This finding might be taken to indicate that the followers did not experience much control over the effects that their actions produced. Such an interpretation would be in line with studies that showed the perceived timing of action effects to depend on causal beliefs about having control (Desantis et al., 2011; Haering and Kiesel, 2012), and the impact of causal beliefs for the processing of temporal delays in general (Greville et al., 2013). By contrast, it is less clear why leaders did not show intentional binding for the follower's action effects even though they clearly showed binding for the follower's action itself (as indicated by the lower F-TAI estimates for the leader as compared to the follower). It seems tempting to explain this null effect by assuming that the explicit knowledge of follower's causing the tone counter-acted intentional binding for the leader. A possible alternative explanation, however, is that the follower's action effects were merely too far removed temporally (Haggard et al., 2002; but see Humphreys and Buehner, 2009). Alternatively, or in addition, intentional binding might have been reduced by the fact that several events—the leader's effect tone and the follower's response—intervened between the leader's action and the followers' action effects.

The notion that action effects produced by another agent at one's command that are far removed from one's own prior actions are associated with reduced agency is interesting in the light of real-world scenarios involving the chain of command, such as in organizational hierarchies or in military decision making. It would be interesting to assess whether individuals higher up in the chain of command do indeed feel less agency for the actions committed by those lower down the chain, and whether those lower down the chain also feel less agency for the *consequences* of their actions when they are made in response to a leader's signal. The results from Experiment 1 of the present experiment seem to suggest that the greatest feeling of agency will be felt by the individual whose direct signal leads to the critical action. However, these ideas remain highly speculative and given that the present results do not allow for any firm conclusions regarding these points, or indeed the general idea of agency for actions taking place after many intervening steps, we will concentrate on the effects that were obtained for the L-ATI and the F-TAI in the following discussion.

A further interesting aspect of the data concerns the participants' responses to the *ad-hoc* questionnaire. Here, leaders described their follower's action by and large in task-related terms, i.e., as responses to the leader's signal. It thus seems as if the leaders construed the follower action indeed as an action effect of their actions which might have been promoted intentional binding for such social action effects. We will get back to this point in the following General Discussion.

## General discussion

Two experiments investigated sense of agency for social action effects in a task in which participants either had the role of a leader or the role of a follower in an action sequence. The leader pressed a key to start off the sequence and produced an effect tone after a variable action-tone interval. This effect tone served as a go signal for the second participant (follower) who pressed his or her key as quickly as possible in response. In Experiment 2, but not in Experiment 1, the second keypress also triggered an effect tone. In different blocks of trials, the participants estimated the duration of three intervals: the L-ATI, the F-TAI, and (in Experiment 2) the F-ATI. Leaders judged the L-ATI and the F-TAI consistently shorter than their followers across both experiments, representing intentional binding for both intervals for the leader. Intentional binding, in turn can be seen as a pre-reflective component of sense of agency for the corresponding action effects (Moore and Obhi, 2012), i.e., the effect tone for the L-ATI and the follower action for the F-TAI.

The observation that the leader showed intentional binding (relative to the follower) not only for his or her own effect tone but also for the response of the follower, suggests that intentional binding does indeed occur when another person's action follows one's own action. The finding of intentional binding for such social action effects extends previous reports on the role of social action effects for effect-based action control (Pfister et al., 2013), by showing that such effects are not only included in action control but may also shape perception similarly to action effects in the physical environment. This notion is also mirrored in the questionnaire data where leaders described the follower response mainly in terms of reacting to the leader's signal.

It should be noted, however, that several factors in the employed design clearly worked in favor of finding binding effects for the leader role. One of these factors becomes evident when considering the exact operationalization of the two roles: Whereas the leader obviously could choose freely when to start the action sequence, the follower did not have this free “when” choice (for a general framework of “when” choices as compared to “what” and “whether” choices, see Brass and Haggard, 2008). Even though some results indicated comparable binding for free-choice and forced-choice responses (Wenke et al., 2009), other findings suggested that at least free “what” choices may promote intentional binding (Barlas and Obhi, 2013). It could thus be argued that the observed binding for social action effects mainly emerged because of the free choice component of the leader's task. Support for this speculation comes from recent findings that indicated the impact of free action choices on effect anticipations to mainly apply to situations in which action-effect relations are somewhat variable (Pfister et al., 2010; Pfister and Kunde, 2013)—and such a variability is clearly present for any type of social action effect due to reduced contingency and contiguity as compared to effects in the physical environment.

Furthermore, the leader's intentional binding of social action effects might also have been boosted by feelings of having power over the follower's behavior. Indeed, power priming has been shown to affect intentional binding, with low power priming decreasing intentional binding as compared to high power priming (Obhi et al., 2012). Whether or not the leaders actually experienced notable feelings of power over the follower's actions cannot be judged from the present experiments, but investigating the impact of power on the perception of social action effects seems to be a promising field for further inquiry.

The present observation of intentional binding for social action effects is also in line with studies that targeted brain activations for participants who performed interdependently on leader-follower tasks. For instance, Chaminade and Decety (2002) employed positron-emission tomography (PET) during a task in which participants moved a circle on a screen in two different conditions. In the leader condition, their own circle was followed by a second circle that was allegedly moved by somebody else, whereas in the follower condition, they were to follow computer-generated movements of the second circle that was said to be moved by another person. Leading and following gave rise to differential activity within the right intraparietal sulcus, a region that has often been associated with sense of agency (e.g., Farrer and Frith, 2002; Spengler et al., 2009). Although Chaminade and Decety did not asses any direct or indirect measures of sense of agency, their results could partly be seen as mirroring sense of agency for social action effects in the leader role, similar to the binding effects observed in our experiments.

The present results are only a first step toward understanding sense of agency for social action effects—a topic that clearly awaits further investigation. This investigation would ideally target sense of agency for social actions with various implicit and explicit measures and at the same time relate these measures to how social action effects are integrated in human motor control in general. A further interesting topic seems to be the impact of unexpected action effects on sense of agency in social settings relative to non-contingent action-effect relations in the physical world (Moore et al., 2009a; Wenke et al., 2009; Sidarus et al., 2013). Indeed, the possibility and problem-spaces relating to agency for actions in social contexts is largely unexplored and there are many exciting opportunities for further research.

In conclusion, social roles like being a leader or a follower while performing a task together have an impact on one's sense of agency, as intentional binding as a pre-reflective component of sense of agency occurs more strongly in the leader than in the follower. Most importantly, our results show that sense of agency does not only occur for physical effects in the environment, but also for social action effects, i.e., predictable actions of other agents.

### Conflict of interest statement

The authors declare that the research was conducted in the absence of any commercial or financial relationships that could be construed as a potential conflict of interest.
